# Duplication and divergence: New insights into *AXR1* and *AXL* functions in DNA repair and meiosis

**DOI:** 10.1038/s41598-020-65734-2

**Published:** 2020-06-01

**Authors:** Marina Martinez-Garcia, Nadia Fernández-Jiménez, Juan L. Santos, Mónica Pradillo

**Affiliations:** 1 Departamento de Genética, Fisiología y Microbiología. Facultad de Biología, Universidad Complutense de Madrid, Madrid, 28040 Spain; 2 Present Address: Department of Genetics, Blavatnik Institute, Harvard Medical School, Boston, MA 02115 USA

**Keywords:** Cell biology, Plant sciences

## Abstract

Rubylation is a conserved regulatory pathway similar to ubiquitination and essential in the response to the plant hormone auxin. In *Arabidopsis thaliana*, AUXIN RESISTANT1 (AXR1) functions as the E1-ligase in the rubylation pathway. The gene *AXR1-LIKE* (*AXL*), generated by a relatively recent duplication event, can partially replace *AXR1* in this pathway. We have analysed mutants deficient for both proteins and complementation lines (with the *AXR1* promoter and either *AXR1* or *AXL* coding sequences) to further study the extent of functional redundancy between both genes regarding two processes: meiosis and DNA repair. Here we report that whereas AXR1 is essential to ensure the obligatory chiasma, AXL seems to be dispensable during meiosis, although its absence slightly alters chiasma distribution. In addition, expression of key DNA repair and meiotic genes is altered when either AXR1 or AXL are absent. Furthermore, our results support a significant role for both genes in DNA repair that was not previously described. These findings highlight that AXR1 and AXL show a functional divergence in relation to their involvement in homologous recombination, exemplifying a duplicate retention model in which one copy tends to have more sub-functions than its paralog.

## Introduction

Ubiquitination is a post-translational modification pathway that affects a wide variety of processes and is highly conserved among eukaryotes^1–3^. Through this process, the small peptide ubiquitin (UBQ) is covalently conjugated to target proteins, altering their stability (by the 26 S proteasome) and activity^4,5^. The attachment of UBQ is orchestrated by a cascade of three specialized enzymes: a UBQ-activating enzyme (E1), a UBQ-conjugating enzyme (E2) and a UBQ-ligase protein (E3)^6–11^. There are a number of UBQ-like modifiers that utilize similar chemical reactions for covalent ligation to their substrates, such as NEURONAL PRECURSOR CELL EXPRESSED DEVELOPMENTALLY DOWN-REGULATED8 (NEDD8) in animals and fission yeast, known as RELATED TO UBIQUITIN (RUB) in plants. While NEDD8 is encoded by a single copy gene in animals, there are three *RUB* genes in *Arabidopsis thaliana*^12^, where *RUB1* and *RUB2* encode UBQ-RUB fusion proteins^13^.

RUB closely resembles UBQ and uses a separate but similar E1-E2-E3 biochemical pathway for activation, conjugation, and ligation. In Arabidopsis, the *E1 C-TERMINAL-RELATED1* (*ECR1*) gene encodes the C-terminal RUB E1 subunit, whereas two paralogous genes, *AUXIN RESISTANT1* and *AXR1-LIKE* (*AXR1* and *AXL*, respectively) encode the N-terminal subunits. Following activation, RUB1 CONJUGATING ENZYME1 (RCE1) and RING-BOX 1 (RBX1) act as RUB-conjugating enzyme E2 and RUB-ligase E3, respectively^14–16^. Rubylation mainly acts as a regulator of cullin-RING E3 UBQ ligases (CRLs). RUB conjugation stabilizes cullins and produces a change of these E3 UBQ ligases that allows the efficient transfer of UBQ to proteins for degradation^17^. Only a few non-cullin substrates have been recently identified, but the biological relevance of these modifications is not yet known (reviewed in Schwechheimer, 2018)^18^.

Rubylation regulates a wide range of cellular processes in Arabidopsis, including cell cycle progression, light response, vegetative growth, embryo development, and auxin signaling^19–23^. In this context, although *AXR1* expression is enriched in tissues with high rates of cellular division, in which the importance of DNA repair is critical, the connection of rubylation with DNA damage response has not currently been established in plants^24,25^. Nevertheless, several publications have described the importance of this pathway in controlling how cells react to DNA breaks in mice and human cell cultures^26–28^.

In Arabidopsis, rubylation is also involved in homologous recombination (HR) during meiosis, the specialised cell division that generates gametes^29^. HR is a key process during this division, since it ensures bivalent formation. Plants deficient in AXR1 display problems in the formation of the synaptonemal complex (SC), the tripartite structure that intimately connects homologous chromosomes during some stages of prophase I, and also changes in the position of crossovers (COs), the reciprocal DNA exchanges that link homologous chromosomes and ensure their correct segregation during the first meiotic division. The mislocalisation of COs in *axr1* leads to the presence of univalents and unbalanced segregations that are the origin of aneuploidies in the gametes^29^. These errors are probably due partly to a deregulation of modular cullin RING ligase 4 (CRL4) whose meiotic targets have not been characterised. The effect of rubylation (also known as neddylation) and CRL4 on synapsis and CO formation has also been described in *Caenorhabditis elegans*^30,31^.

AXR1 and AXL share 80% of amino acid identity. They have redundant functions in RUB activation and show similar biochemical activity *in vitro*^32–35^. Because of the key importance of rubylation in plant growth and development, *axr1 axl* double mutants are usually lethal at embryonic or early seedling stage. However, whereas *axl* plants present wild-type (WT) phenotypes, *axr1* plants are dwarf and exhibit multiple growth defects, small flowers and short siliques^29,33,36^. For a long time, it was thought that AXR1 and AXL were functionally equivalent, since Dharmasiri and colleagues^33^ demonstrated that the phenotype of *axr1* plants could be restored by overexpression of *AXL*. Later, Hotton and colleagues^35^ proved that the two genes are not equivalent: *AXL* cannot replace *AXR1* when it is expressed at comparable levels to *AXR1* in an *axr1* background. Trying to delve into this issue, we have analysed the consequences of the absence of AXR1 and AXL, focusing our attention on the HR process, essential for genome maintenance and meiotic chromosomal segregation. Results presented here demonstrate that both proteins are not functionally equivalent during meiotic recombination, although both are essential for efficient DNA repair. Furthermore, the corresponding mutants (*axr1* and *axl*) exhibit a significant reduction in *HEI10* expression in meiotic tissues. This is a gene homolog of *HUMAN ENHANCER OF INVASION CLONE10* which encodes a SUMO (Small Ubiquitin-like Modifier) E3 ligase necessary for the formation of the majority of COs in Arabidopsis^37^. We conclude that the functional divergence of AXR1 and AXL is clearly more pronounced in meiotic than in somatic recombination, although AXR1 also plays a more predominant role than AXL in this process. Altogether, these findings suggest that both proteins have probably evolutionary adjusted their preferences for different interactors depending on specific cellular stages or environments.

## Results

### *axr1* and *axl* mutants show differences in fertility

**Figure 1:**
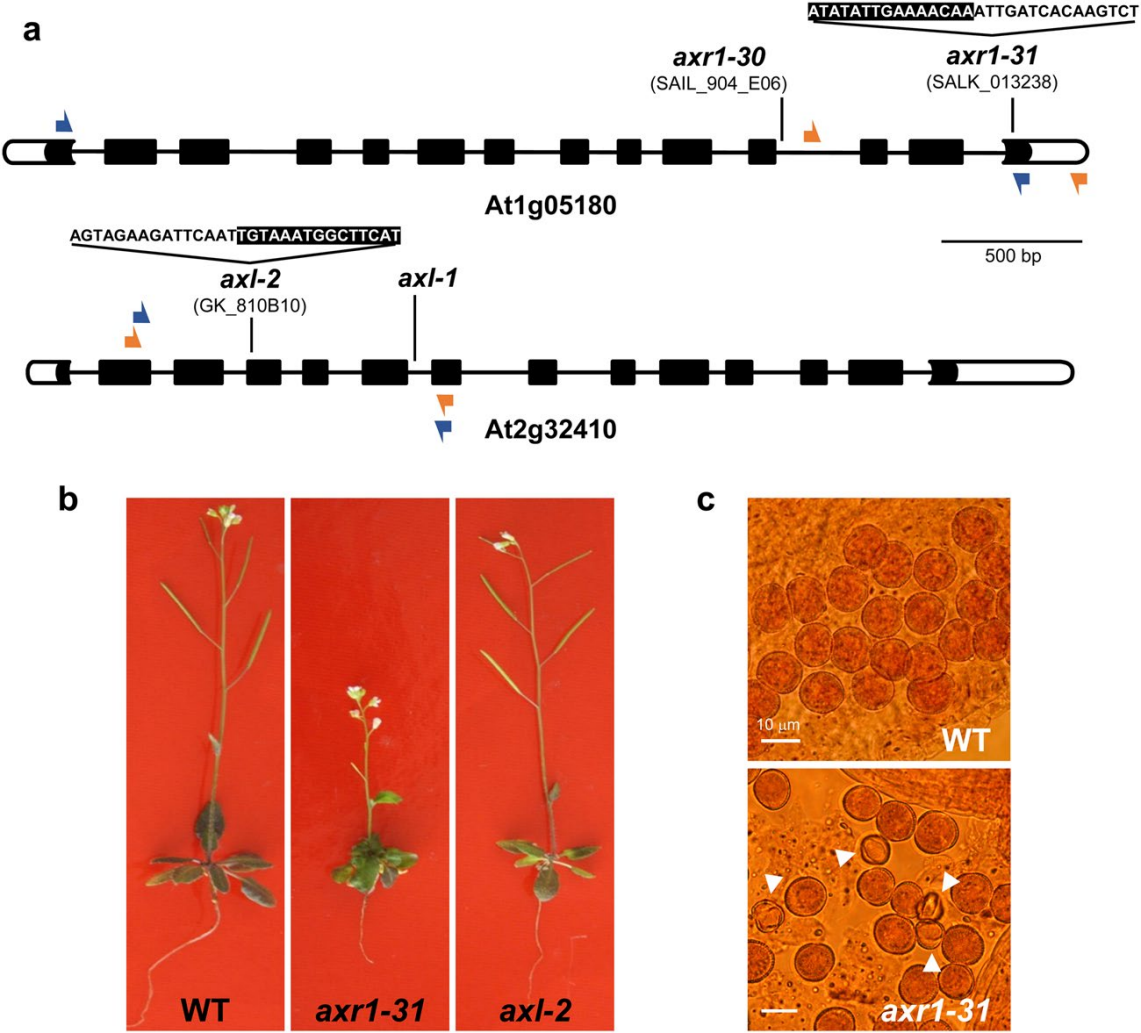
Insertion sites and phenotypic features of mutant lines for the genes *AXR1* and *AXL*. (**a**) *AXR1* (At1g05180) and *AXL* (At2g32410) gene structures and localisation of mutant alleles. The T-DNA is located 3,386 nt and 686 nt downstream from the ATG in *axr1-31* and *axl-2*, respectively, and its sequence is represented by white letters on black background. The mutant alleles *axr1-30* and *axl-1* were described in previous publications^33,35^. Exons: black boxes; introns: lines joining the boxes; 5′ and 3′ UTRs: white boxes. Orange and blue arrows represent primers used for genotyping and RT-PCR, respectively. **(b)** Representative pictures of WT, *axr1-31* and *axl-2* plants (40 days after sowing). **(c)** Squashed anthers from WT and *axr1-31* plants containing mature pollen grains. Arrowheads point to abnormal (small) pollen grains in *axr1-31*.

RT-PCR analyses revealed that neither full-length transcripts of *AXR1* nor *AXL* were produced in the T-DNA insertion lines *axr1-31* (SALK_013238) and *axl-2* (GK-818B10) mutants, respectively (Fig. 1A, Supplementary Fig. S1). However, in agreement with previous observations^19,24^, homozygous *axl* plants were indistinguishable from WT plants in terms of germination, vegetative growth, and flowering time, whereas homozygous plants for *axr1* alleles displayed severe growth alterations and developmental defects. These plants also showed leaves with irregular shape and slightly jagged edges, and reduced height (maximum ~20 cm in mature plants versus 50 cm in WT plants) (Fig. 1B). In accordance with the phenotype displayed by other *axr1* mutants^29^, *axr1-31* showed an increase of irregular pollen grains, smaller than WT, with irregular shape, shrunken and collapsed (26.72%, 144/539 in *axr1-31*; 1.00%, 6/600 in WT ; χ^2^_1_ = 164.206, p < 0.001; Fig. 1C) and a severe reduction in the number of seeds *per* silique (5.67 ± 1.25 in *axr1-31*; 48.53 ± 1.17 in WT; two-tailed Mann Whitney U-test p < 0.001). Siliques were also shorter than in WT (4.40 ± 0.35 mm in *axr1-31*; 12.47 ± 0.22 mm in WT; two-tailed Mann Whitney U-test p < 0.001).

### AXR1 and AXL are not functionally equivalent during meiosis

**Figure 2:**
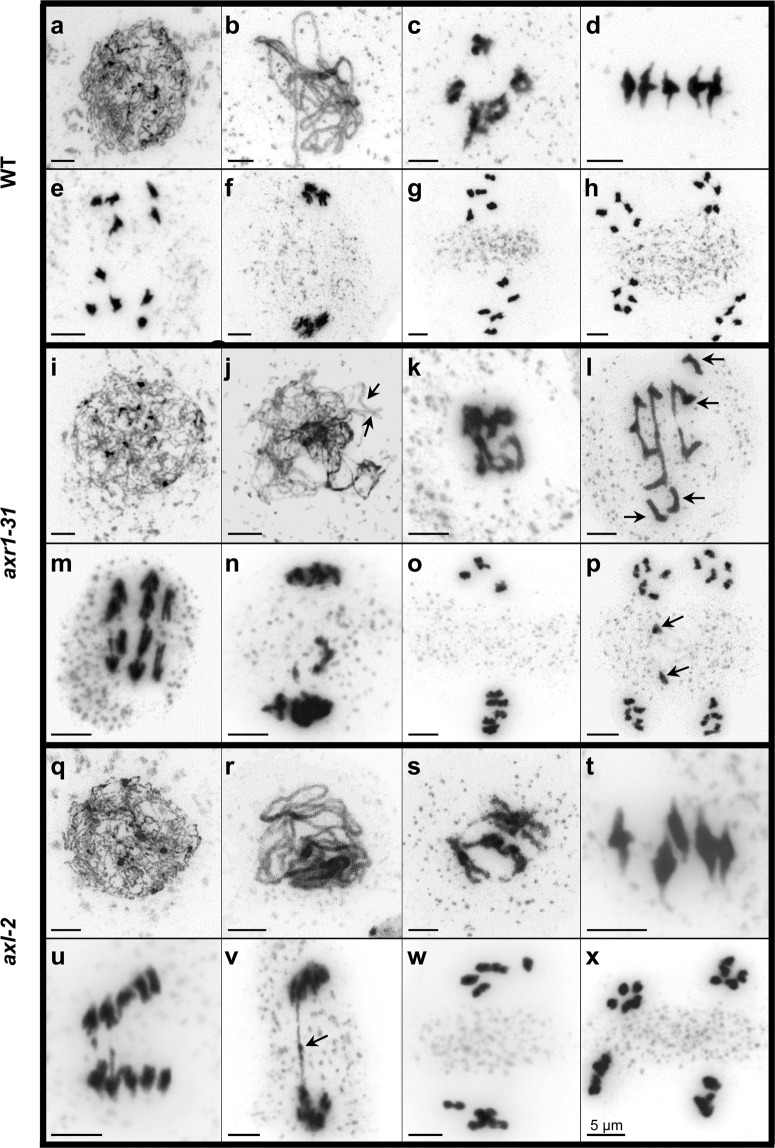
Meiosis in PMCs from WT (**a**–**h**), *axr1-31* (**i**–**p**), and *axl-2* (**q**–**x**). (**a**,**i**,**q**) Leptotene meiocyte **(b**,**j**,**r)** Pachytene meiocyte. Arrows indicate incomplete synapsis in *axr1-31*. **(c**,**k**,**s)** Diakinesis. **(d**,**l**,**t)** Metaphase I. Univalents are only present in *axr1-31* (arrows). **(e**,**m**,**u)** Anaphase I. **(f**,**n**,**v)** Telophase I. A bridge connecting the two poles can be distinguished in *axl-2* (arrow). **(g**,**o**,**w)** Metaphase II. In *axr1-31* the nuclei are unbalanced. **(h**,**p**,**x)** Telophase II. There are chromatids in the organelle band (arrow).

*AXR1* and *AXL* genes present high expression levels in flower stage 9, when male meiosis occurs^38^. It has been reported that reduced fertility in *axr1* mutants is related to alterations in meiosis^29^, but to date no observations of meiosis in pollen mother cells (PMCs) of *axl* mutants had been reported, perhaps because they display apparent normal fertility^33^. Figure 2 shows a comparison of different meiotic stages in PMCs of WT and *axr1-31* and *axl-2* plants, as representative examples of *axr1* and *axl* mutants, respectively. No differences among the genotypes were found at leptotene stage (Fig. 2A,I,Q), but *axr1* showed incomplete synapsis in pachytene cells (n = 48; Fig. 2B,J). Problems in synapsis were confirmed using antibodies against the central element protein ZYP1 and the axial element-associated protein ASY1 (Supplementary Fig. S2). Fully synapsed bivalents were observed in *axl* at this stage (Fig. 2R). Depletion of the function of either AXR1 or AXL produced loosened chromatin at diakinesis (100%, n = 15 and 27.3%, n = 11, respectively; Fig. 2C,K,S). This feature persisted even up to metaphase I in some *axr1* cells (Fig. 2L). Only *axr1* displayed univalents at metaphase I (1.43 ± 0.13 pairs of univalents *per* cell, n = 72 vs 0 in WT and *axl*, Fig. 2L) that lead to abnormal chromosome segregations at anaphase I and unbalanced nuclei at second meiotic division (86.4%,  n = 44; Fig. 2M–P). This shortage in bivalent formation observed in *axr1-31* was similar to that described for other *axr1* alleles (1.0 and 1.3 pair of univalents *per* cell in *axr1-12* and *axr1*-SAIL_904_E06^29^). Although five bivalents were always present in *axl* (Fig. 2T), some anaphase I and telophase I meiocytes showed chromatin bridges connecting the two nuclei (33%, n = 54, Fig. 2U,V). No evidence of chromosome fragmentation was observed in any mutant, suggesting that all programmed double-strand breaks (DSBs) produced at early prophase I were repaired (Fig. 2L–P,T–X).

**Figure 3:**
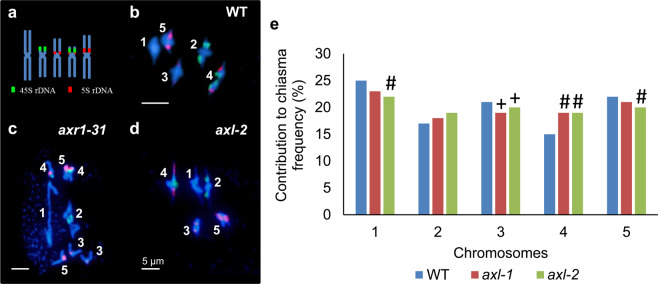
Cytogenetic analysis of metaphase I cells by FISH. Probes against 45 S (green) and 5 S (red) rDNA were used to analyse the behaviour of specific chromosomes. (**a**) Ideogram showing the location of the probes on the chromosomes. **(b)** Metaphase I cell from WT. **(c)** Metaphase I cell from *axr1-31*. **(d)** Metaphase I cell from *axl-2*. E. Contribution of each chromosome pair to the total chiasma frequency. ^+^p ≤ 0.01; ^#^p ≤ 0.001.

Arabidopsis chromosomes can be easily identified by FISH according to their morphology and the position of 45 S and 5 S rDNA sequences (Fig. 3A–D). To further analyse the meiotic phenotype produced by the *axl* mutation and determine the possible existence of variations in the behaviour of the different chromosomes, we conducted an in-depth cytological analysis of metaphase I meiocytes to estimate meiotic recombination by measuring chiasmata *per* cell. As it has been mentioned before, unlike the situation in *axr1* (Fig. 3C), we did not observe univalents in *axl* (Fig. 3D). Table 1 shows the mean chiasma frequencies *per* cell, *per* chromosome and *per* arm in two *axl* mutant lines (*axl-1* and *axl-2*), and WT plants. Despite dissimilarities in individual chromosomes compared to WT, differences in the overall mean chiasma frequencies were not detected among the different genotypes (Table 1; Fig. 3E). In WT plants, the submetacentric/metacentric group of chromosomes formed by chromosomes 1, 3 and 5 normally accounts for 21–25% of the total chiasmata *per* cell, whereas small acrocentric chromosomes 2 and 4 contribute to 15–17% of the total chiasmata^39^. We observed significant decreases in the contribution of chromosome 3 to the total chiasma frequency in *axl-1*, and of chromosomes 1, 3 and 5 in *axl-2*. In contrast, in both mutants there was an increase in the contribution of chromosomes 2 and 4 (only statistically significant in chromosome 4) (Table 1; Fig. 3E).

**Table 1:** Mean chiasma frequencies *per* cell, *per* chromosome and *per* arm in *axl* mutants.

	Chiasmata *per*	Bivalents										Total	
		1		2		3		4		5			
		s	l	s	l	s	l	s	l	s	l	C	n
WT	arm	—	—	0.61	1.14	0.90	1.26	0.48	1.01	0.97	1.30	10.20	69
	chromosome	2.52 25%		1.75 17%		2.16 21%		1.49 15%		2.28 22%			
*axl-1*	arm	—	—	0.86	0.90	0.92	1.02	0.90	0.96	0.94	1.20	10.04	49
	chromosome	2.34 23%		1.76 18%		1.94^a^ 19%		1.86^b^ 19%		2.14 21%			
*axl-2*	arm	—	—	0.88	0.97	0.86	1.08	0.84	1.01	0.96	1.09	9.94	89
	chromosome	2.25^c^ 22%		1.85 19%		1.94^d^ 20%		1.85^e^ 19%		2.05^f^ 20%			

s: short arm; l: long arm; C: mean cell chiasma frequency; n: total number of cells analysed *per* genotype. The percentages represent the chromosome contribution to the total chiasma frequency. In chromosome 1, due to the absence of FISH signals, it is not possible to distinguish the short arm from the long arm. ^a^p = 0.0146, ^b^p = 0.0001, ^c^p = 0.0008, ^d^p = 0.0098, ^e^p < 0.0001, ^f^p = 0.0007 (Two-tailed Mann Whitney U-test).

**Figure 4:**
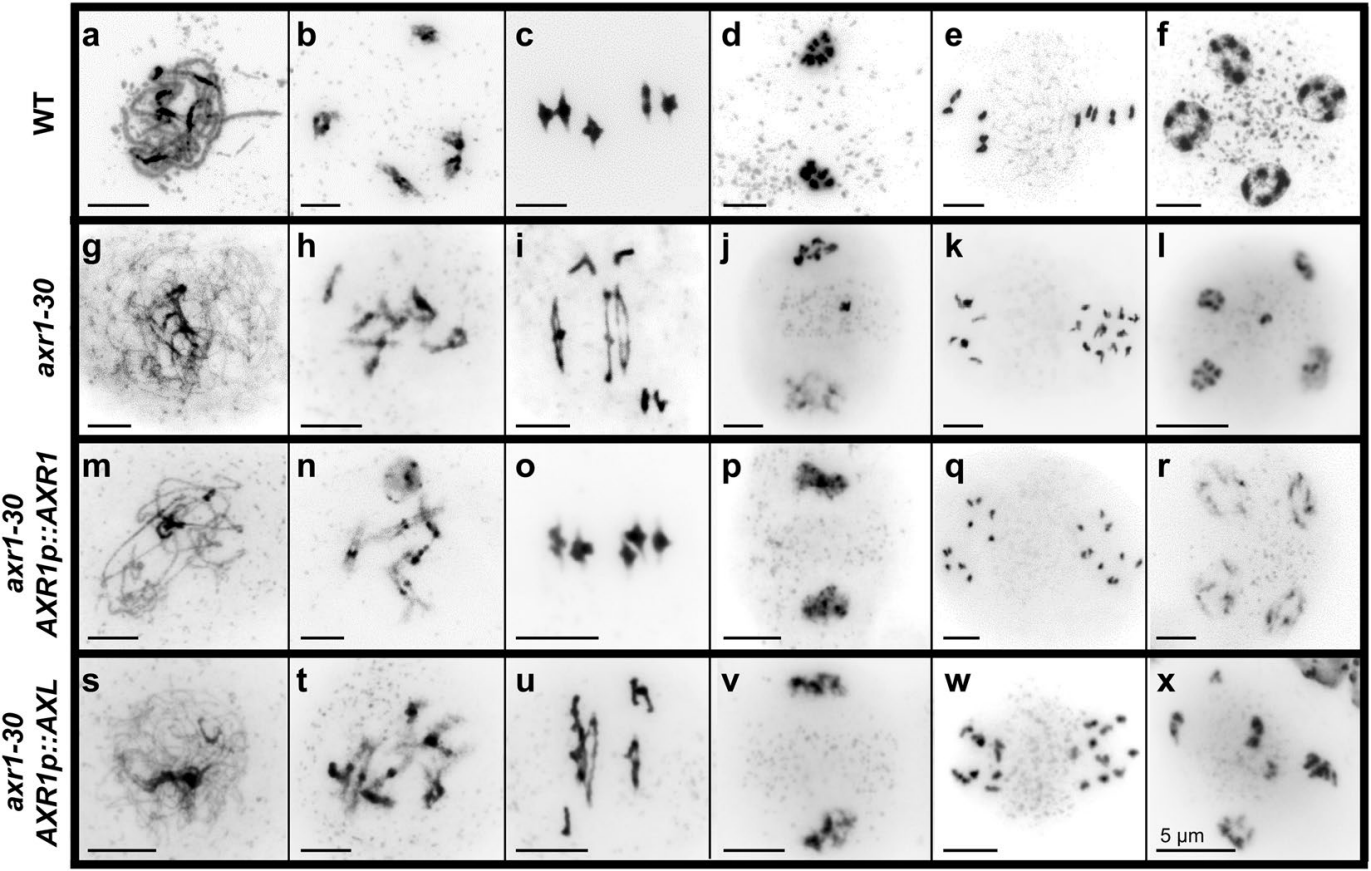
Meiosis in PMCs from WT plants (**a**–**f**), *axr1-30* (**g**–**l**), *axr1-30 AXR1p::10MYC-AXR1* (**m**–**r**), and *axr1-30 AXR1p::10MYC-AXL* (**s**–**x**). (**a**,**g**,**m**,**s**) Early prophase I. **(b**,**h**,**n**,**t)** Late prophase I. **(c**,**i**,**o**,**u)** Metaphase I. **(d**,**j**,**p**,**v)** Prophase II. **(e)** Metaphase II. **(k**,**q**,**w)** Anaphase II. **(f**,**l**,**r**,**x)** Tetrad.

We have obtained additional evidence that highlights the different functions of AXR1 and AXL during meiosis using complementation lines of the mutation *axr1-30*. This mutation (1.57 ± 0.19 pairs of univalents *per* cell, n = 33; Fig. 4G–L, Supplementary Fig. S3) produces the same defects (in terms of univalent frequency) as *axr1-31* (1.43 ± 0.13; two-tailed Mann Whitney U-test p = 0.582). Its complementation with an expression cassette containing the *AXR1* promoter, a sequence for an N-terminal 10xMYC epitope tag, and the *AXR1* coding sequence (*AXR1p::10MYC-AXR1*)^35^, resulted in bivalent frequency restoration (n = 24; Fig. 4M–R, Supplementary Fig. S3). On the other hand, the complementation of *axr1-30* with a similar cassette but containing the *AXL* coding sequence (*AXR1p::10MYC-AXL*)^35^ did not restore the WT bivalent frequency (0.72 ± 0.11 pairs of univalents *per* cell, n = 32; Fig. 4S–X, Supplementary Fig. S3; two-tailed Mann Whitney U-test p = 0.002), although it increased significantly the bivalent frequency in comparison to *axr1-30* (two-tailed Mann Whitney U-test p = 0.0012). The restoration was not produced despite the fact that the expression levels of both genes (*AXR1* and *AXL*) were controlled by the same promoter (*AXR1p*).

### AXR1 and AXL are involved in DNA repair

During the cell cycle, DSBs are produced by endogenous or exogenous sources, but during meiosis, recombination events are initiated by programmed DSBs^40^. Indeed, many of the genes involved in meiotic recombination have a role during somatic recombination and mutants with altered CO frequency have also reduced ability to repair DNA damage produced from a variety of exogenous sources. For this reason, we decided to determine the DNA repair capability of *axr1* and *axl* mutants in relation to that of WT plants in response to the following agents: γ rays, mitomycin C (MMC), cisplatin (CDDP), and UV-C radiation.

**Figure 5:**
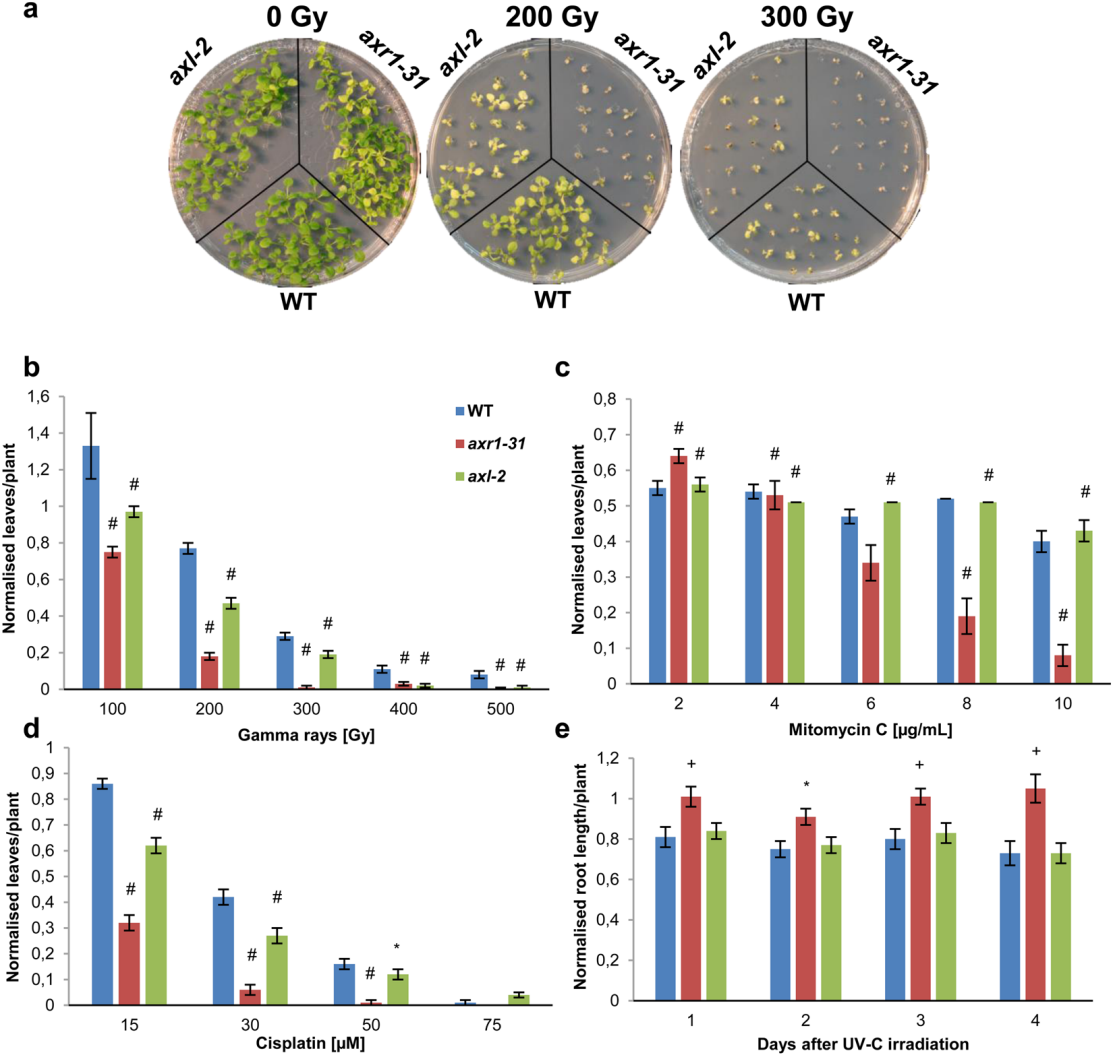
DNA damage sensitivity assays in *axr1-31* and *axl-2*. (**a**) Representative images of WT, *axr1-31* and *axl-2* seedlings after exposure to 0, 200 and 300 Gy. **(b)** Number of leaves *per* plant in WT, *axr1-31*, and *axl-2* seedlings after the exposure to different doses of gamma radiation. **(c)** Number of leaves *per* plant in WT, *axr1-31*, and *axl-2* seedlings after the exposure to different concentrations of MMC. **(d)** Number of leaves *per* plant in WT, *axr1-31*, and *axl-2* seedlings after the exposure to different concentrations of CDDP. **(e)** Root length *per* plant in WT, *axr1-31*, and *axl-2* seedlings during different days after the exposure to 500 J/m^2^ of UV-C light irradiation. All the results were normalised to standard grown conditions. *p ≤ 0.05; ^+^p ≤ 0.01; ^#^p ≤ 0.001.

Gamma rays are a powerful inductor of complex DSBs that can be repaired through different pathways, one of them being HR^41^. Both *axr1-31* and *axl-2* mutants were hypersensitive to this agent, though *axr1-31* to a greater extent, and showed a reduced mean number of true leaves *per* plant in the different doses applied (Fig. 5A,B; Supplementary Table S2). Similar results were obtained analysing the relative dry weight *per* plant (Supplementary Fig. S4; Table S3). MMC mainly forms crosslinks (CLs) between complementary strands of DNA. These lesions are highly cytotoxic, and their repair is primarily triggered by stalled replication forks and depends on HR^42–44^. Only *axr1-31* plants displayed hypersensitivity after MMC treatment in comparison with WT plants at higher concentrations (>6 μg/mL) (Fig. 5C and Supplementary Fig. S5; Table S2). Data corresponding to relative dry weight showed similar behaviour (Supplementary Fig. S4; Table S3). CDDP differs from MMC since it is a chemotherapy drug that induces higher relative levels of intra-strand CLs versus inter-strand CLs in the DNA^45,46^. These adducts, which affect only one strand of the DNA, are mainly removed by nucleotide excision repair (NER). However, HR is also critical to manage CDDP toxicity, probably because this agent can eventually produce inter-strand CLs^47,48^. As in the case of gamma rays, both *axr1-31* and *axl-2* were hypersensitive to CDDP, *axr1-31* being even more affected by the treatment (Fig. 5D, Supplementary Fig. S4; Tables S2 and S3).

UV-C radiation mainly causes a photo-chemical effect in adjacent thymines that leads to their dimerization^49^. Photoreactivation is thought to be the major DNA repair pathway for these pyrimidine dimers in plants^50^. Enzymes involved in this pathway remove DNA lesions by absorbing light. Repair without a light source includes NER and other DNA repair pathways such as base excision repair (BER) or mismatch repair (MMR)^50,51^. By covering the plants with aluminium foil after irradiation we avoided photorreactivation, favouring the repair by NER and the other DNA repair pathways mentioned above. Unlike the previous treatments, no hypersensitivity response was detected in any of the mutants. Moreover, *axr1-31* plants exhibited a slightly better response than the control (Fig. 5E; Supplementary Table S2).

### The expression of genes involved in HR is disturbed in *axr1*

Given the differences between *axr1* and *axl* in their behaviour during meiotic recombination and DNA repair, we considered evaluating the expression of several genes by qRT-PCR. Specifically, we analysed transcription levels of *AXR1*, *AXL*, *Topoisomerase II* (*TOPII*, involved in chromosome condensation^52^ and interlock resolution^53^), and sixteen key genes for HR (Supplementary Table S1). Since the mutations *axr1* and *axl* affect auxin response, it is important to note that several of the analysed genes present an auxin-responsive element AuxRE (Auxin-Responsive Element) in their promoters: *ATM, ATR, BRCA2A, BRCA2B, MLH3, MND1, MSH4, MSH5, RAD51C, RMI1*, and *XRI1*^54,55^ (Promomer, http://bar.utoronto.ca/ntools/cgi-bin/BAR_Promomer.cgi). We have conducted qRT-PCR parallel analyses on samples from flower buds and young seedlings to determine whether or not the variations in the gene expression levels are meiosis-specific.

**Figure 6:**
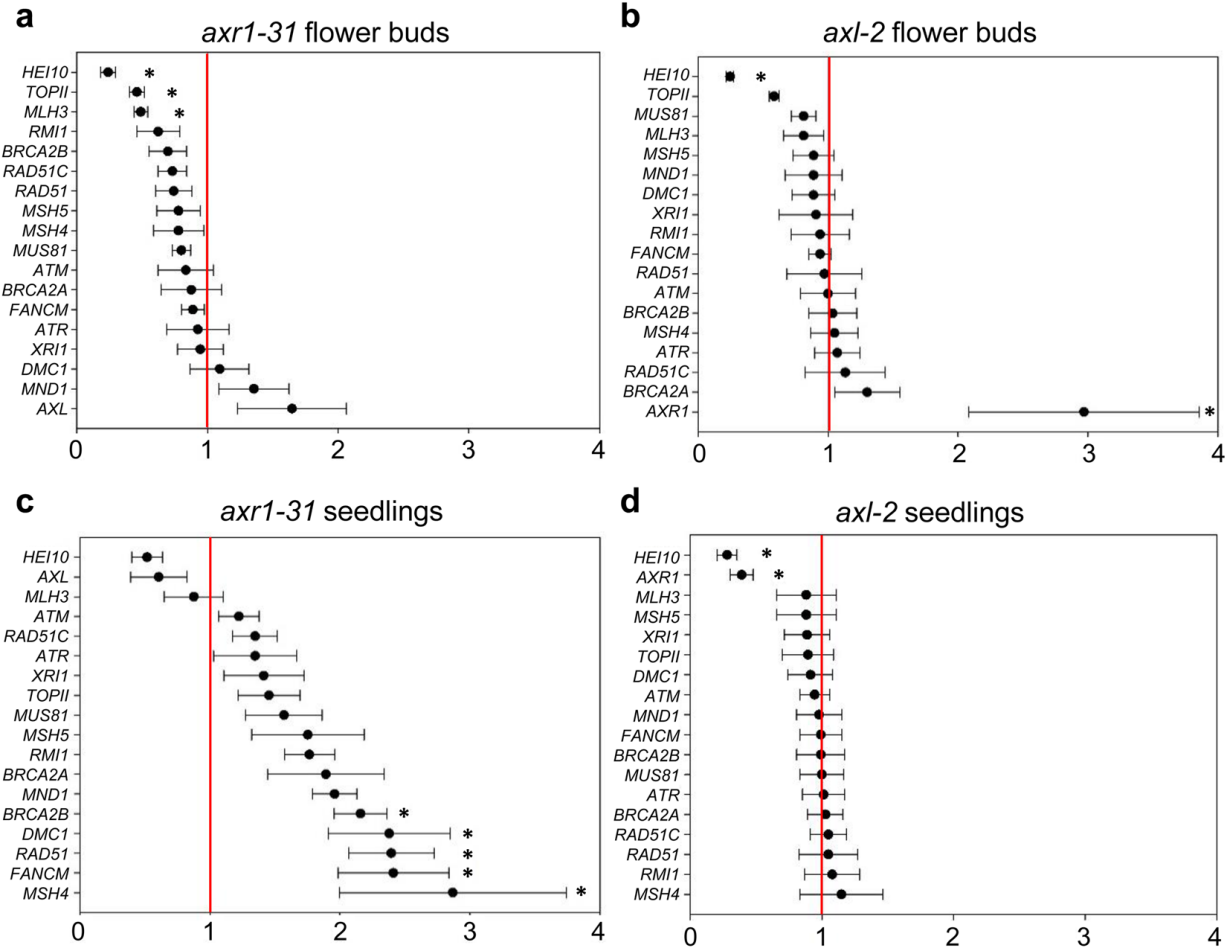
Analysis of the expression of 18 genes by qRT-PCR in meiotic tissue and seedlings from *axr1-31* (**a**,**c**) and *axl-2* (**b**,**d**). The values represent the fold change variation compared to WT levels (line in 1). The asterisks indicate either significant up-regulation (>2) or down-regulation (<0.5).

A noteworthy result was the overexpression of each corresponding paralogous gene in flower bud samples from both mutants: *AXL* expression was up-regulated in *axr1* flower buds, and *AXR1* was in *axl* (Fig. 6A,B), revealing a possible functional relationship between both genes. The observed overexpression seems to be meiosis-specific, since it was not detected in seedlings, in which even *AXR1* was down-regulated in *axl-2* samples (Fig. 6D). The reduced expression of *HEI10* in flower bud samples from both mutants (and in seedlings from *axl-2*) was also relevant (Fig. 6). This gene encodes a conserved meiotic ubiquitin E3 ligase required for the formation of most COs in Arabidopsis^37,56^. *TOPII* was also down-regulated in flower bud samples from both mutants (although not statistically significantly in *axl-2*) (Fig. 6A,B). In the case of samples obtained from seedlings it is noteworthy that the expression levels of the genes analysed were normal in *axl-2* (in which only *HEI10* and *AXR1* were under-expressed), whereas several genes were up-regulated in *axr1-31*: *BRCA2B, DMC1, RAD51, FANCM* and *MSH4* (Fig. 6C,D).

## Discussion

Duplicated genes have played a critical role in the evolution of all major eukaryotic linages, especially in plants. Their preponderance is consequence of the high rate of duplication occurrence along evolution together with preferential retention of paralogous genes (retention bias). After duplication, maintenance of a perfect equivalency in gene function is unusual and most models of duplicate retention assume various degrees of functional redundancy because of changes in function, expression, or interactions with other gene products^57^. Among Angiosperms, the existence of distinguishable *AXR1* and *AXL* genes seems to be limited to Arabidopsis species. In other plant species there are either well preserved copies, virtually identical, or only one *AXR1* gene^35^. Why are two functional copies necessary in the Arabidopsis genome? Hotton and colleagues^35^ suggested that the two corresponding proteins could interact with different factors. Alternatively, AXR1 and AXL could have different roles during certain cellular environments or developmental stages. Here we report new evidence that point in this direction.

Despite *AXR1* and *AXL* are highly expressed in the ninth stage of flower development, *axr1* and *axl* mutants showed differences in fertility that could be related to conspicuous differences in meiotic chromosome behaviour (Fig. 2). Meiocytes from plants deficient in AXR1 did not display complete synapsis during pachytene stage and presented a high frequency of univalents at metaphase I that led to abnormal chromosome segregations at anaphase I and unbalanced second division nuclei^29^ (Fig. 2I–P). On the other hand, as expected from their full fertility, meiosis was normal in *axl* plants, with the exception of the presence of chromosome bridges at anaphase I (Fig. 2Q–X). Although *axl* and WT plants exhibited a similar mean cell chiasma frequency, there were slight differences in the contribution of each chromosome to the total chiasma frequency (Fig. 3E; Table 1). Particularly, in comparison with the situation observed in WT cells, the decrease in the contribution of long chromosomes 1, 3, and 5 was compensated by an increase in the contribution of the acrocentric chromosomes (2 and 4). These last chromosomes are the smallest and bear the nucleolar organising regions (NORs) (Fig. 3A). Under altered CO distribution circumstances, chromosomes 2 and 4 could have more chances to recombine due to the physical proximity of the homologous pairs through their nucleolar domains^58,59^.

Jahns and colleagues^29^ reported that the conspicuous decrease in the frequency of bivalents showed by *axr1* mutants was not due to a decrease in CO frequency but rather due to changes in CO distribution. The phenotype we observed in *axl* might be reflecting slight variations in the distribution of COs, although not as drastic as those observed in *axr1*. In this context, altered chiasma distribution in *axr1-31* and *axl-2* might be related to impaired expression of the meiotic E3 ligase *HEI10*, since this gene was down-regulated in both genetic backgrounds (Fig. 6A,B). Supporting this idea, HEI10 (as well as its orthologues RNF212 and ZIP3^37,60,61^) is postulated to participate in designating HR intermediates as COs and has been described as a dosage-sensitive regulator of class I COs during Arabidopsis meiosis^56^. We also detected decreased expression levels of *TOPII* (Fig. 6A,B), which has been involved in chromosome condensation and meiotic interlock resolution in *A. thaliana*^53^. In other organisms, mutations affecting this gene are related to alterations in chromosome condensation, defects in chromosome segregation, and reduced strength of CO interference^62–64^. Abnormal chromosome condensation, altered CO distribution and chromosome segregation problems were observed in *axr1* meiocytes and also in *axl* to a lesser extent (Fig. 2), suggesting that part of their phenotypes could be due to an impaired activity of TOPII. Nevertheless, neither the absence of AXR1 nor AXL appear to alter the expression of the other meiotic genes tested in our experiments, even to those with an auxin-related sequence motif (AuxRE) motif on their promoters.

We have obtained more results indicating that AXR1 and AXL are not functionally equal in meiosis, the former playing a more active role than the later. The presence of the cassette *AXR1p::10MYC-AXR1* was capable to complement the strong *axr1-30* mutant phenotype in bivalent formation, while *AXR1p::10MYC-AXL* was only able to do it partially: univalents were still present, but with a lower frequency (Fig. 4, Supplementary Fig. S3). This result is in line with that obtained in the analyses by qRT-PCR. *AXR1* displayed a three-fold change compared to WT levels in flower bud samples from *axl-2* and *AXL* was also overexpressed in flower bud samples from *axr1-31* (Fig. 6). One possible explanation is that the increased expression of *AXR1* in *axl-2* is sufficient to ensure normal meiosis, while the presence of a greater amount of *AXL* does not allow it in *axr1-31*. These findings indicate that the functions of AXR1 and AXL on meiotic recombination are not redundant.

Most proteins that participate in meiotic recombination are also active during somatic DNA damage repair. However, their somatic functions might differ from their meiotic roles through interactions with different factors or post-translational modifications. Presumably, according to the results discussed above, AXR1 and AXL diverged in relation to their meiotic recombination functions. In this study we find for the first time a role for both rubylation E1 subunits AXR1 and AXL. Nevertheless, as far as their role in DNA repair, although with subtle differences, the function of both genes appears to be more redundant. Both *axr1-31* and *axl-2* plants showed hypersensitivity to gamma-rays and CDDP, although only *axr1-31* plants were hypersensitive to MMC (Fig. 5; Supplementary Table S2). In the latter, AXR1 might act as a back-up in *axl-2* mutant background, whereas its presence is not enough to avoid the hypersensitivity phenotype displayed after the treatment with the other DNA damage agents. These results suggest that although both AXR1 and AXL are involved in DNA repair they are not equally important in the repair of the different CLs produced by the treatments. One possible explanation is that AXL has a more prominent role in the repair through NER, since this is the main repair pathway for intra-strand CLs created by CDDP, whereas AXR1 would have a more direct involvement in HR repair, the process required to remove the inter-strand CLs formed by MMC. In agreement with this idea, genes involved in HR were up-regulated in *axr1-31* seedlings in relation to WT, whereas they presented normal levels in *axl-2* seedlings (Fig. 6D). In this context, it has been reported that expression of *AXR1* after exposure to a mix of bleomycin and MMC is slightly higher than in normal conditions, whereas *AXL* levels do not change^65^. CDDP also induces inter-strand CLs (although to a lesser extent than intra-strand CLs) and this could be the reason why both mutants were hypersensitive to this agent.

The results corresponding to the UV radiation experiment did not indicate hypersensitivity in any of the mutants (Fig. 5E; Supplementary Table S2). Furthermore, *axr1-31* showed some tolerance to the treatment. In previous studies, the rubylation pathway has been indirectly linked to UV-C sensitivity through the ubiquitin-mediated degradation of photomorphogenic factors, interconnecting light signalling and DNA repair^66^. It is tempting to speculate that the efficiency of other DNA repair pathways different from HR could be improved in this genetic background in which HR is deficient, although future experiments should be performed to test this hypothesis. Because of the lethality of the *axr1 axl* double mutant, it is not possible to conduct sensitivity assays in this background. Nevertheless, the results obtained indicate that the two proteins could have non-overlapping functions and cannot complement each other, presenting more defects when AXR1 is absent. The lethality of the double mutant might even reflect that both proteins are involved in parallel DNA repair pathways.

Indirect proofs about the relationship between rubylation and DNA repair have been obtained in mouse models and human cells. In these materials the combination of either MMC or CDDP and MLN4924, the inhibitor of NEDD8/RUB-activating enzyme, synergistically enhances the cytotoxicity of both drugs through increased DNA damage^67,68^. Moreover, the N-terminal neddylation of histone H4 in response to DNA damage in mammalian cells suggests that this modification could break inter-nucleosome interactions facilitating the access to the repair machinery^26^. Neddylation has also been shown to regulate DSB pathway choice and the balance between non-homologous end-joining (NHEJ) and HR in somatic DNA repair by inhibiting CtIP-mediated DNA end resection^27^ and promoting Ku ubiquitination and its release from damage sites^69^. In *A. thaliana*, the complex CUL4-DDB1-DDB2 (CULLIN4 together with DNA DAMAGE BINDING PROTEINS 1 and 2), a substrate of AXR1 rubylation pathway, ubiquitylates a high variety of proteins during DNA repair by NER in an ATR-dependent manner^70^. CUL4 levels also increase after genotoxic damage^65^. In relation to meiotic recombination, CUL4 is apparently involved in the deregulation of CO localisation observed in *axr1* mutants^29^. However, *cul1* and the hypomorphic *cul3a/3b* mutants, defective for cullins that are also rubylated, display normal meiotic behaviour^29^. Interestingly, CUL1 partner ASK1 (Arabidopsis SKP1-like1) is required to ensure proper chromosome condensation, synapsis and chromosome segregation in male Arabidopsis meiosis^71^. Our results showing DNA repair deficiencies in mutants for RUB-activating enzyme confirm more directly the connection between rubylation and DNA repair in plants. However, further studies with mutants defective for other proteins involved in rubylation will be necessary to reveal a more mechanistic view of the link between this post-translational modification and both somatic and meiotic HR.

Based on all the results presented here it seems that the asymmetry in the pattern of functional partitioning between these paralogs is different during meiosis and DNA repair. These genes are predicted to have duplicated after the more recent whole-genome duplication event (α)^72^. After duplication, random genetic drift might lead to an uneven contribution of the two copies, with AXR1 taking on a leading role, this being more pronounced in meiosis. This model seems to be intermediate among Angiosperms species, in which either there are two identical copies in sequence, and therefore with the same function, or one of the copies has been lost. The existence of distinguishable AXR1 and AXL proteins could influence their preferences for different interactors depending on the developmental stage or the cellular environment. This difference could in turn lead to some specialisation (such as the repair of one of the types of CLs or involvement in a particular DNA repair pathway). This situation has been also described for other Arabidopsis genes such as *OSD1* (*OMISSION OF SECOND DIVISION 1*), a gene essential to enter into the second meiotic division, and its paralog *UVI4* (*UV-B-INSENSITIVE 4*), without a meiotic function but required for the cell cycle^73,74^. The study of these genes in species close to Arabidopsis, not yet characterized, might shed some light on the possible consequences of these divergences.

## Methods

### Plant material

Several transfer DNA (T-DNA) mutant lines defective for the genes *AXR1* (At1g05180) and *AXL* (At2g32410) were analysed (Fig. 1A). The lines *axr1-31* (SALK_013238) and *axl-2* (GK_818B10) were identified by “Signal T-DNA Express Arabidopsis Gene Mapping Tool” (http://signal.salk.edu/cgi-bin/tdnaexpress) and obtained from Nottingham Arabidopsis Stock Centre (NASC). Numbers were assigned to each of these new alleles in concordance to previous references^33,75,76^. Line *axl-1* was provided in homozygous condition by Prof. Mark Estelle (University of California San Diego, USA). Lines *axr1-30* (SAIL_904_E06)*, AXR1p:10MYC-AXR1* and *AXR1p::10MYC-AXL* (all homozygous for *axr1-30*) were kindly donated by Prof. Judy Callis (University of California Davis, USA). Arabidopsis Col-0 accession was used as a control.

### Growth conditions and plant genotyping

Seeds were sown in pots containing a soil mixture of vermiculite and commercial soil (3:1). Plants were maintained in a growth chamber (temperature 18–20 °C; photoperiod 16 h light/8 h dark).

Genomic DNA was extracted mashing young leaves in extraction buffer (100 mM Tris pH 9.5; 250 mM KCl: 10 mM EDTA). After 10 minutes at 95 °C, samples were diluted in BSA 3%. PCR amplifications of mutant alleles were performed using a specific T-DNA primer and a T-DNA flanking primer (RP). Specific T-DNA primers were: LBa1 (5′TGGTTCACGTAGTGGGCCATCG3′) for SALK lines; and o8409 (5′ATATTGACCATCATACTCATT3′) for the GK line. T-DNA flanking primers used were: 5′TCATGTGGAGAATGGGCTTAC3′ (RP-*axr1-31*) and 5′AAGTATCACCATTGTCGACGG3′ (RP-*axl-2*). WT alleles were amplified with primers LP-*axr1-31* (5′TGTGATTGAATATTGCAGGAGC3′) and RP-*axr1-31*; LP-*axl-2* (5′ TCTTCATCCGCTGATACCATC3′) and RP-*axl-2*.

### Identification of abnormal pollen grains and fertility assessment

Pollen grains were screened using the pollen squash method. Mature flowers were dissected, and the anthers were stained with 2% aceto-carmine (Sigma) and fixed to the slide through a flame. Slides were observed by phase-contrast microscopy. To evaluate fertility, data corresponding to the number of seeds *per* silique and silique length were collected from five randomly selected siliques (once they completely developed but before they had dropped the seeds) belonging to three plants of each genotype.

### Cytological techniques

Flower buds were fixed in Carnoy (60% ethanol, 30% chloroform, 10% acetic acid) at room temperature during at least 24 h and kept at −20 °C until used. Chromosome spreads and fluorescence *in situ* hybridization (FISH) were performed according to the methodology previously described (the most recent version of the protocol is reported in^77^). More than 50 cells *per* genotype were analyzed at each stage. The following DNA probes were used: pTa71 [45 S ribosomal DNA (rDNA), pTa71 of *Triticum aestivum*^78^] and pCT4.2 (5 S rDNA^79^).

Immunolocalisation procedures were conducted as detailed in Armstrong and colleagues^80^, with slight modifications^81^. The primary antibodies used were kindly provided by Prof. Chris Franklin (University of Birmingham): anti-AtASY1 (rat; 1:1,000 dilution), anti-AtZYP1 (rabbit; 1:500).

Slide preparations were observed with an Olympus BX61 microscope equipped with epifluorescence optics. Images were captured with an Olympus DP70 digital camera controlled by DP Controller software version 2.2.1.227 (Olympus).

### DNA damage sensitivity analyses

Seeds were surface sterilized for 5 min in 2.5% sodium hypochlorite and rinsed with sterilized distilled water at least five times. After leaving them overnight at 4 °C, the seeds were plated in plastic Petri plates containing germination media (GM^82^: salt mixture (1×) in 1% sucrose, and 1% agarose) to conduct the genotoxic assays following the protocol reported in Martinez-Garcia and Pradillo (2017)^77^. To test the sensitivity to γ-rays, sterilized seeds were kept overnight in sterile water at 4 °C and then exposed to 0, 100, 200, 300, 400, and 500 Gy doses (2.94 Gy/min) from a ^137^Cs source (IBL 437 C; CIS Bio). After irradiation, seeds were germinated on GM agar medium. Different concentrations for mitomycin C (MMC, Duchefa Biochemie: 2, 4, 6, 8 and 10 µg/mL) and cisplatin [cis-diamminedichloroplatinum (II), CDDP, Sigma: 0, 15, 30, 50, and 75 μM] were also applied. Solutions were prepared and then added directly into MS media after cooling down. Plates were maintained under standard growth conditions (temperature 22–25 °C; photoperiod 16 h light/8 h dark) until analysis. The effects of the DNA damage agents on plant growth (number of true leaves) were evaluated 12 days (MMC) or 14 days (γ-rays, CDDP) after sowing. For the UV-C damage assay, 5-day-old seedlings grown in vertical square plates were irradiated for 5 minutes with a dose of 500 J/m^2^ from a Sanyo Denki g15T8 lamp. Irradiated plants were grown in dark conditions to avoid photorreactivation, favouring DNA repair by nucleotide excision repair (NER). Root lengths were measured 1, 2, 3, and 6 days after the treatment. Statistical comparison was performed using non-parametric two-tailed Mann Whitney U-test. P-values are available in Supplementary Table S2.

### Gene expression analyses

For reverse transcription PCR (RT-PCR) and quantitative RT-PCR (qRT-PCR) experiments, total RNA was extracted from 10-day-old seedlings and flower buds using RNeasy PlantMini kit (Qiagen) and DNase enzymatic treatment (RNase-Free DNase Set, Qiagen). mRNAs from *AXR1* and *AXL* were retrotranscribed with One Step RT-PCR Kit (Qiagen). *GAPC* (glyceraldehyde-3-phosphate dehydrogenase, cytosolic gene) was used as constitutive control. Primers used in the RT-PCR were: F-AXR1: 5′ATGCAAGCAGTAAAAAGATCCAG3′ and R-AXR1: 5′CTACAATTTCAATAACTGAGAC3′; F-AXL: 5′TGGTAGCAGCACTCAAGGAG3′ and R-AXL: 5′CCGTCACAACCCATTTCACT3′; N-GAPC: 5′CTTGAAGGGTGGTGCCAAGAAGG3′ and C-GAPC: 5′CCTGTTGTCGCCAACGAAGTCAG3′.

qRT-PCR was performed with the Transcriptor First Strand cDNA Synthesis kit and the FastStart TaqMan Probe Master kit (Roche). Primers were designed using the tool *UPL Assay Design Centre* (Roche) and their sequences are listed in Supplementary Table S1. Data were analyzed considering fold variation over a calibrator using the ΔΔCt method and the *ACTIN2* gene as endogenous control. At least three experimental replicates were performed. We considered a fold-change variation significant when there was a minimum of two-fold change compared to the WT levels: RQ higher than 2 or lower than 0.5 and non-overlapping 95% confidence intervals.

### Database resources and bioinformatic tools

The identification of T-DNA insertion lines, analysis of sequences and primer design were performed using databases and tools from National Centre for Biotechnology Information (http://www.ncbi.nlm.nih.gov/), The Arabidopsis Information Resource (http://www.arabidopsis.org/), Nottingham Arabidopsis Stock Centre (http://arabidopsis.info/) and The Salk Institute Genome Analysis Laboratory (http://signal.salk.edu/cgi-bin/tdnaexpress/). Gene interactions were analysed using Promomer (http://bar.utoronto.ca/ntools/cgi-bin/BAR_Promomer.cgi). Statistical analyses were conducted with Microsoft Excel and the software IBM SPSS 22. Data in the text with their mean as measures of center, is followed by ± their standard error as a measure of variability. Statistical comparisons between values were made using non-parametric two-tailed Mann Whitney U-test, whether comparisons between frequencies were made using χ^2^_1_ test. Adobe Photoshop CS4, Microsoft Excel, SigmaPlot, and ImageJ were used for graph and image composition.

## Supplementary information

Supplementary Information.

## Data Availability

All data generated or analysed during this study are included in this published article (and its Supplementary Information files). Biological materials will be shared upon request to the corresponding author.
