# Supplementary material for: Duplication and divergence: New insights into AXR1 and AXL functions in DNA repair and meiosis
Source: Sci Rep. 2020 Jun 1;10:8860. doi: 10.1038/s41598-020-65734-2 (PMC7264244; doi:10.1038/s41598-020-65734-2)
Supplement: Supplementary file 1 — Supplementary Information. [file 41598_2020_65734_MOESM1_ESM.pdf]

Supplementary Information

Duplication and divergence: New insights into *AXR1* and *AXL* functions in DNA repair and meiosis

Marina Martinez-Garcia[1,2], Nadia Fernández-Jiménez[1], Juan L. Santos[1], Mónica Pradillo[1*]

[1]Departamento de Genética, Fisiología y Microbiología. Facultad de Biología. Universidad Complutense de Madrid, Madrid, 28040, Spain.

[2]Department of Genetics, Blavatnik Institute, Harvard Medical School. Boston, MA 02115, USA.

*** Correspondence:**
Mónica Pradillo
pradillo@bio.ucm.es

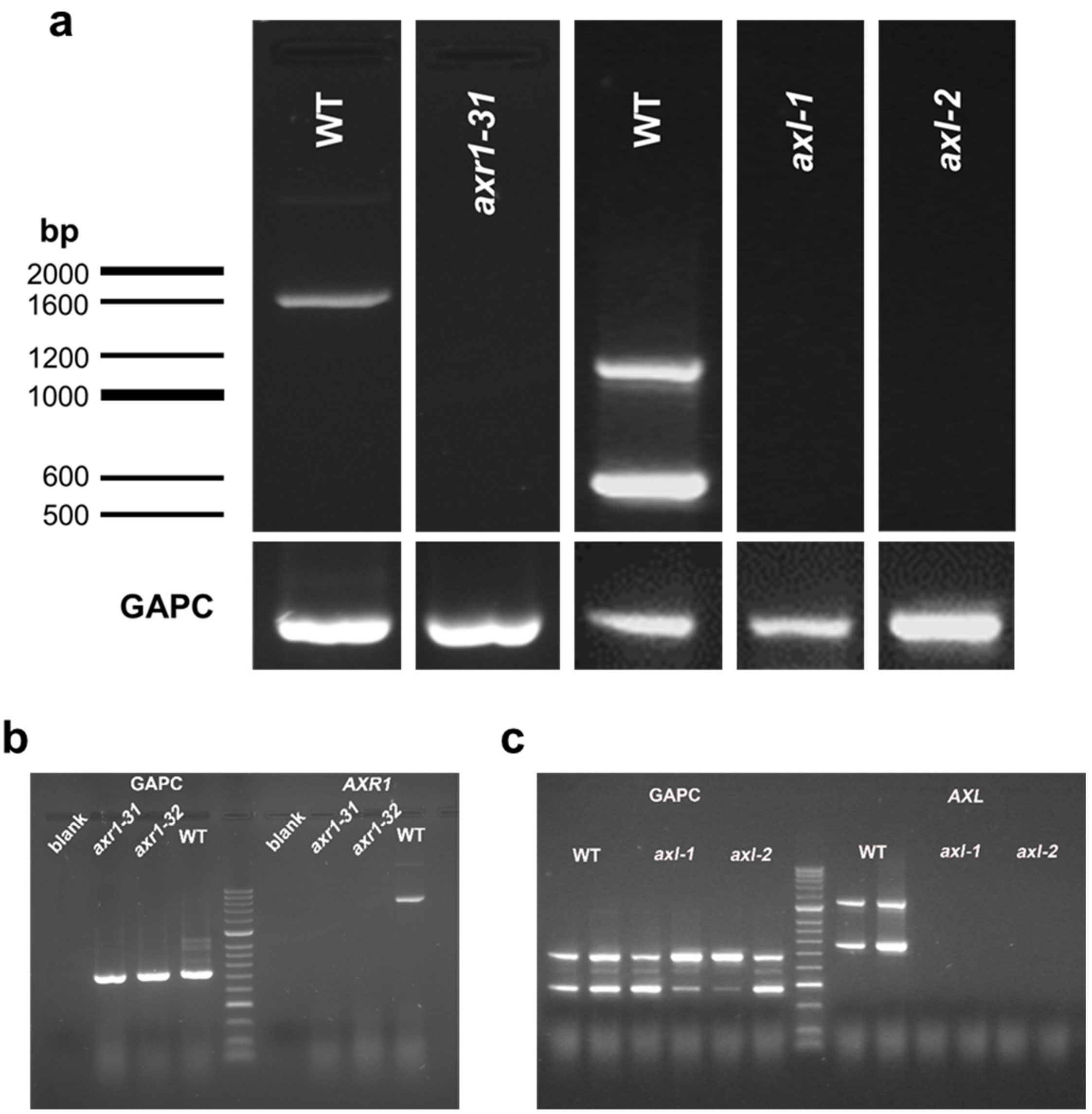

Supplementary Figure 1. Expression analyses of *AXR1* and *AXL* by RT-PCR. (a) Cropped gels showing that neither full-length transcripts of *AXR1* nor *AXL* were produced in *axr1* and *axl* mutants, respectively. The location of the primers is depicted in Figure 1. The WT lanes for both *AXR1* and *AXL* display two bands: one corresponds to the gDNA (3,419 bp for *AXR1* and 1,066 bp for *AXL*) and another corresponds to the cDNA (1,634 bp for *AXR1* and 570 bp for *AXL*). **(b)** Original gel image for *AXR1* expression analysis. **(c)** Original gel image for *AXL* expression analysis.

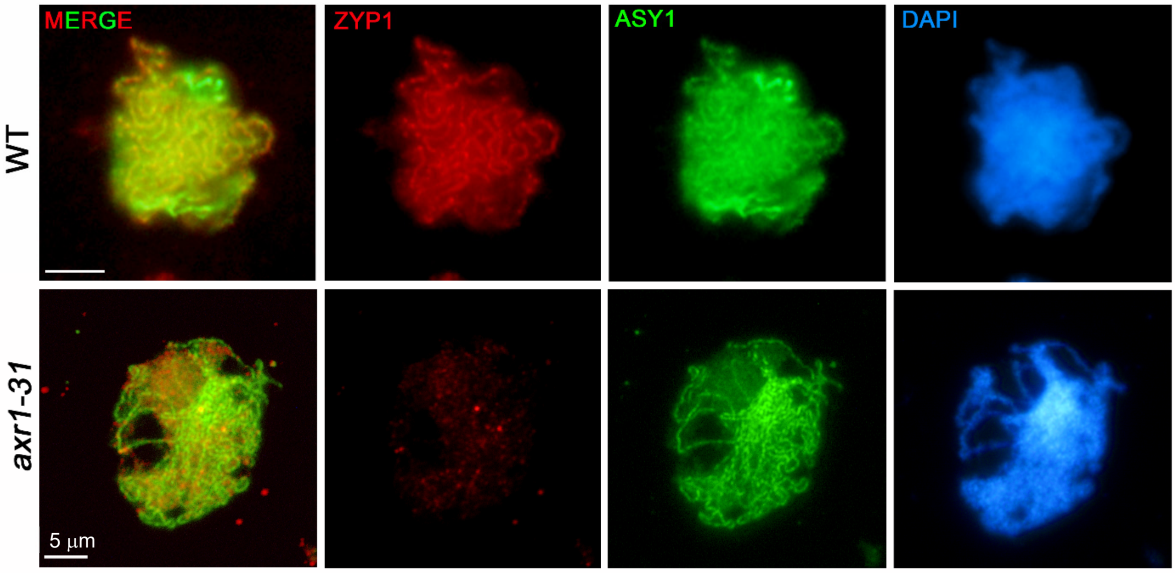

Supplementary Figure 2. Immunolocalisations of ASY1 (axial element-associated protein) and ZYP1 (central element) confirm synapsis defects detected after DAPI staining of the chromosomes in *axr1-31*. Merged and ZYP1 (red), ASY1 (green), and DAPI (blue) images are shown.

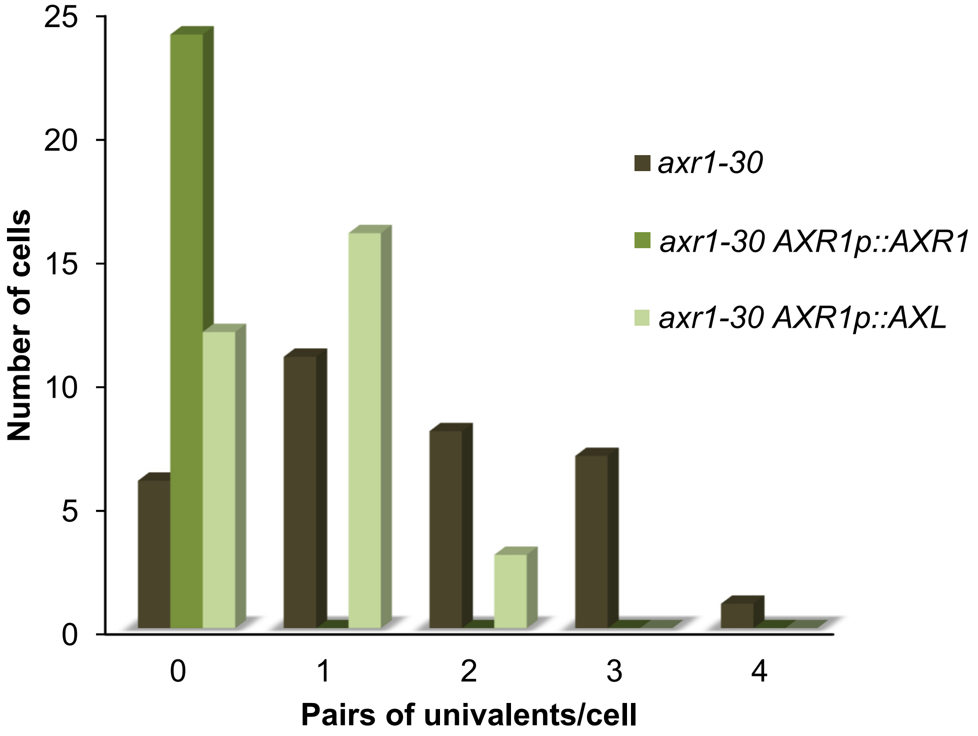

Supplementary Figure 3. Pairs of univalents *per* cell in metaphase I meiocytes from *axr1-30* and *axr1-30* plants complemented with *AXR1p::10MYC-AXR1* or *AXR1p::10MYC-AXL.* Bivalent frequency was restored in *axr1-30 AXR1p::10MYC-AXR1* plants.

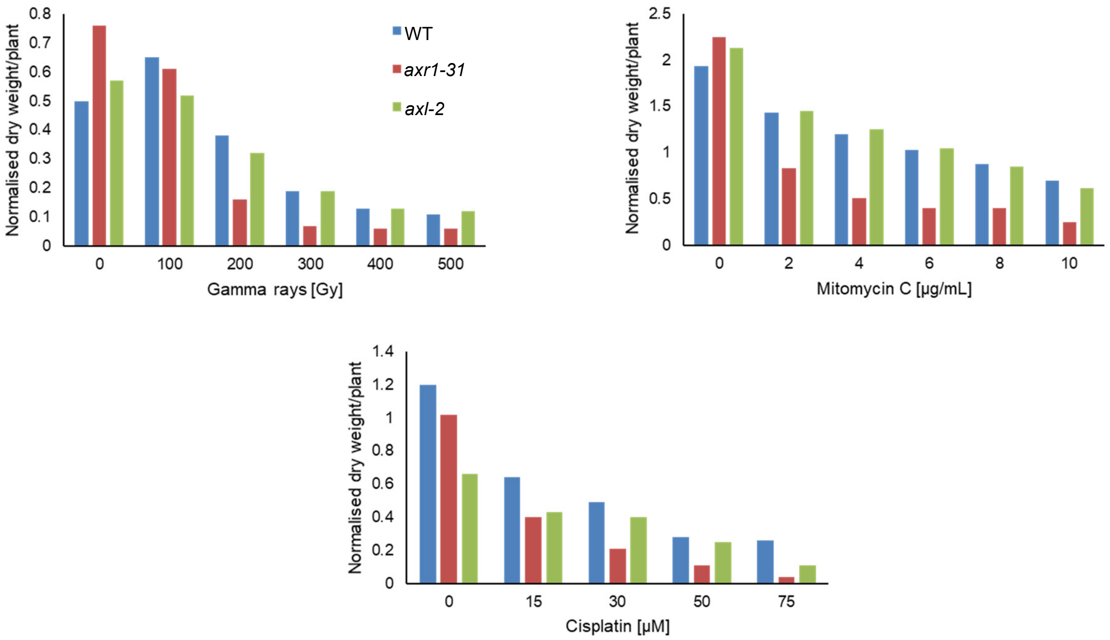

Supplementary Figure 4. Dry weight *per* plant after treatments with genotoxic agents: gamma rays, MMC and CCDP. The data were normalised compared to either untreated seedlings or plants grown on genotoxin-free medium (see Supplementary Table 3).

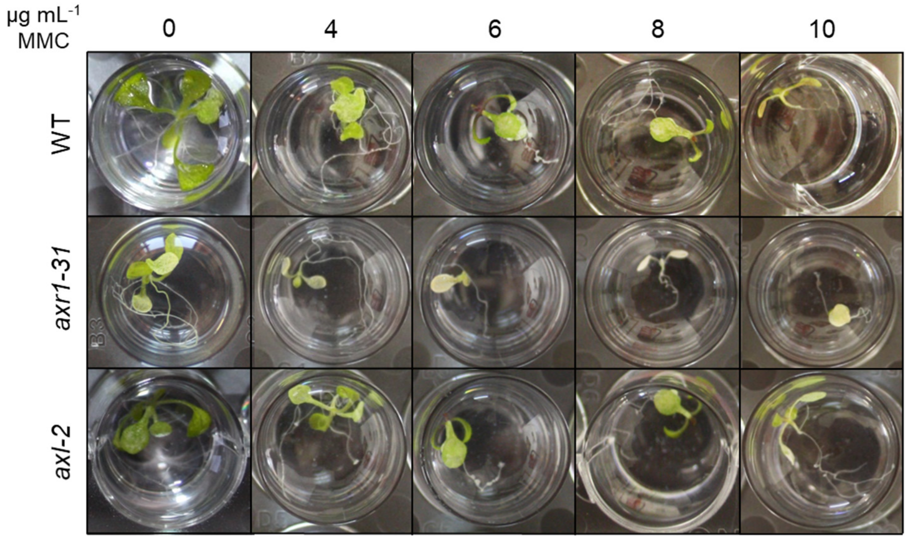

Supplementary Figure 5. Phenotypes of seedlings (WT, *axr1-31*, and *axl-2*) grown on liquid media containing different concentrations of MMC.

Supplementary Table 1. Oligonucleotide sets used for qRT-PCR analyses.

**Gene**	**Sequence 5´→3´**	**UPL**
ATM	AGGGTGGTGAGATGAGAAGC	98
	TCTGTGTCAATTGCGTCTTGT	
ATR	TTCAGCGCCCAAAGAAGA	3
	GGCTTGCAGAGGAATGGATA	
AXL	ATGGCCTGAGCTGAAGAGC	41
	TTGCCGCTATGGTGTTGAG	
AXR1	CCGAGGCTTTGAAGAATCTC	119
	GATTGGCCAACACTCTTCG	
BRCA2A	TTGGTTGGATCCGTGGTC	74
	TCTGCGTTTATGAAGTAGACTGAGTT	
BRCA2B	CACCTTAAAACCCGCAGTG	140
	AGGTGATTTACAAGCACCGATT	
DMC1	TCAACGTTGCTGTCTACATGACT	31
	GACCACCTGCTGGCTTTTT	
FANCM	TCGCGGTAAAAAGCACATTA	143
	GACATCTCTGCTCCCGAAGA	
HEI10	CATGTCCCATTTGTGATCAAGT	151
	TCGGTATGCACTCTTCATCAGT	
MLH3	TCACACTTAATGCGGTTCCA	146
	CTCGAAGAACAGATGGAGGAA	
MND1	TGCGAAAGACAAGATTGGAA	33
	AAGTTTCTGGCGAACACTCC	
MSH4	TCGATACTCTAGACCTGAACTAACAGA	159
	TGCTTTCTAGGATGGGATGC	
MSH5	GATCGCCGTCTCAATGCTA	156
	ATTTCAGTGTCTCCCGCAAT	
MUS81	GATATGTACCCAACGCTTTTGTC	29
	CTTCTTGCGCCGAGACAT	
RAD51	CATGCCACCACAACAAGG	91
	ACATGGCGAGCTTATCACTTTAC	
RAD51C	TCAACTAGCGCTTGCTTTAGG	54
	AATACAGAATGACTCGGTTGGTG	
RMI1	TTCCAGTTGTTTCTCGCTTGA	157
	TTGCGACGATCTGTTCATTT	
TOPII	GAGCAAAGAAGAAGGCTCCA	134
	GCCGTTGTGCTAGATCCAA	
XRI1	CTCAGCTTATAGAAGGGTTTTCAGA	34
	CCACTCTTCAGCGTACAGATCA	
ACTIN2 (control)	CCGCTCTTTCTTTCCAAGC	30
	CCGGTACCATTGTCACACAC	

Supplementary Table 2. DNA damage sensitivity assays in *axr1-31* and *axl-2* plants. The relative number of leaves (or root length) *per* plant was normalised in relation to either untreated seedlings or plants grown on genotoxin-free medium. * $p \leq 0.05$; + $p \leq 0.01$; # $p \leq 0.001$.

Treatment	Dose	Average			Mann Whitney U-test		
**Gamma rays (leaves/plant)**	**Gy**	**Col**	***axr1-31***	***axl-2***	**Col/ *axr1-31***	**Col/ *axl-2***	***axr1-31/ axl-2***
	100	1.33 ± 0.18	0.75 ± 0.03	0.97 ± 0.03	< 0.001# (2364.00)	< 0.001# (3210.00)	< 0.001# (2736.00)
	200	0.77 ± 0.03	0.18 ± 0.02	0.47 ± 0.03	< 0.001# (720.00)	< 0.001# (1847.00)	< 0.001# (1384.00)
	300	0.29 ± 0.02	0.01 ± 0.01	0.19 ± 0.02	< 0.001# (1476.00)	< 0.001# (2917.00)	< 0.001# (2216.00)
	400	0.11 ± 0.02	0.03 ± 0.01	0.02 ± 0.01	< 0.001# (3587.00)	< 0.001# (3250.00)	0.129 (4357.00)
	500	0.08 ± 0.02	0.00 ± 0.00	0.01 ± 0.01	< 0.001# (2695.00)	< 0.001# (3605.00)	0.074 (3619)
**Mitomycin C (leaves/plant)**	**µg/mL**	**Col**	***axr1-31***	***axl-2***	**Col/ *axr1-31***	**Col/ *axl-2***	***axr1-31/ axl-2***
	2	0.55 ± 0.02	0.64 ± 0.02	0.56 ± 0.02	< 0.001# (78.00)	< 0.001# (152.00)	< 0.001# (156.00)
	4	0.54 ± 0.02	0.53 ± 0.04	0.51 ± 0.00	< 0.001# (306.00)	< 0.001# (0.00)	< 0.001# (240.00)
	6	0.47 ± 0.02	0.34 ± 0.05	0.51 ± 0.00	0.231 (684.00)	< 0.001# (160.00)	0.402 (720.00)
	8	0.52 ± 0.00	0.19 ± 0.05	0.51 ± 0.00	0.001# (480.00)	< 0.001# (0.00)	0.001# (480.00)
	10	0.40 ± 0.03	0.08 ± 0.03	0.43 ± 0.03	< 0.001# (357.50)	< 0.001# (324.00)	< 0.001# (300.00)
**Cisplatin (leaves/plant)**	**µM**	**Col**	***axr1-31***	***axl-2***	**Col/ *axr1-31***	**Col/ *axl-2***	***axr1-31/ axl-2***
	15	0.86 ± 0.02	0.32 ± 0.03	0.62 ± 0.03	< 0.001# (380.00)	< 0.001# (776.00)	< 0.001# (836.50)
	30	0.42 ± 0.03	0.06 ± 0.02	0.27 ± 0.03	< 0.001# (729.00)	< 0.001# (781.00)	< 0.001# (830.50)
	50	0.16 ± 0.02	0.01 ± 0.01	0.12 ± 0.02	< 0.001# (3120.00)	0.034* (2460.50)	< 0.001# 2097.00)
	75	0.01 ± 0.01	0.00 ± 0.00	0.04 ± 0.01	0.150 (2448.00)	0.135 (2136.00)	0.009+ (2124.00)
**UV-C light (root length/plant)**	**Days after treatment**	**Col**	***axr1-31***	***axl-2***	**Col/ *axr1-31***	**Col/ *axl-2***	***axr1-31/ axl-2***
	1	0.81 ± 0.05	1.01 ± 0.05	0.84 ± 0.04	0.005+ (99.00)	0.652 (244.00)	0.036* (127.00)
	2	0.75 ± 0.04	0.91 ± 0.04	0.77 ± 0.04	0.012* (111.00)	0.835 (255.00)	0.074 (139.00)
	3	0.80 ± 0.05	1.01 ± 0.04	0.83 ± 0.05	0.005+ (99.00)	0.621 (242.00)	0.013* (112.00)
	6	0.73 ± 0.06	1.05 ± 0.07	0.73 ± 0.05	0.003+ (34.00)	0.930 (103.00)	0.002+ (27.00)

Supplementary Table 3. Dry weight *per* plant after treatments with different genotoxic agents: gamma rays, MMC and CCDP. The data were normalised in relation to either untreated seedlings or plants grown on genotoxin-free medium.

Gamma rays			
Gy	Col	*axr1-31*	*axl-2*
0	0.50	0.76	0.57
100	0.65	0.61	0.52
200	0.38	0.16	0.32
300	0.19	0.07	0.19
400	0.13	0.06	0.13
500	0.11	0.06	0.12

Mitomycin C			
µg/mL	Col	*axr1-31*	*axl-2*
0	1.93	2.25	2.13
2	1.43	0.83	1.45
4	1.20	0.51	1.25
6	1.03	0.40	1.05
8	0.88	0.40	0.85
10	0.70	0.25	0.62

Cisplatin			
µM	Col	*axr1-31*	*axl-2*
0	1.20	1.02	0.66
15	0.64	0.40	0.43
30	0.49	0.21	0.40
50	0.28	0.11	0.25
75	0.26	0.04	0.11
